# Plethysmography System to Monitor the Jugular Venous Pulse: A Feasibility Study

**DOI:** 10.3390/diagnostics11122390

**Published:** 2021-12-18

**Authors:** Antonino Proto, Daniele Conti, Erica Menegatti, Angelo Taibi, Giacomo Gadda

**Affiliations:** 1 Department of Cybernetics and Biomedical Engineering, VSB–Technical University of Ostrava, 17. Listopadu 2172/15, 70800 Ostrava, Czech Republic; antonino.proto@vsb.cz; 2 Department of Physics and Earth Sciences, University of Ferrara, Via Saragat 1, 44122 Ferrara, Italy; daniele.conti@edu.unife.it; 3 Vascular Diseases Center, Department of Translational Medicine, University of Ferrara, Via Aldo Moro 4, 44124 Ferrara, Italy; mngrce@unife.it; 4 Section of Ferrara, National Institute for Nuclear Physics (INFN), Via Saragat 1, 44122 Ferrara, Italy; gadda@fe.infn.it

**Keywords:** jugular venous pulse, internal jugular vein, capacitive strain gauge, plethysmography, electrocardiography, wearable device

## Abstract

Cerebral venous outflow is investigated in the diagnosis of heart failure through the monitoring of jugular venous pulse, an indicator to assess cardiovascular diseases. The jugular venous pulse is a weak signal stemming from the lying internal jugular vein and often invasive methodologies requiring surgery are mandatory to detect it. Jugular venous pulse can also be extrapolated via the ultrasound technique, but it requires a qualified healthcare operator to perform the examination. In this work, a wireless, user-friendly, wearable device for plethysmography is developed to investigate the possibility of monitoring the jugular venous pulse non-invasively. The proposed device can monitor the jugular venous pulse and the electrocardiogram synchronously. To study the feasibility of using the proposed device to detect physiological variables, several measurements were carried out on healthy subjects by considering three different postures: supine, sitting, and upright. Data acquired in the experiment were properly filtered to highlight the cardiac oscillation and remove the breathing contribution, which causes a considerable shift in the amplitude of signals. To evaluate the proper functioning of the wearable device for plethysmography, a comparison with the ultrasound technique was carried out. As a satisfactory result, the acquired signals resemble the typical jugular venous pulse waveforms found in literature.

## 1. Introduction

Nowadays, there is a worldwide increasing interest in the topic of circulatory oscillations assessment, because of the recognized importance in the diagnosis and prevention of cardiovascular diseases [1].

Human brain drainage is investigated in diagnosing heart failure through the jugular venous pulse (JVP) waveform, a descriptor of the venous outflow within the internal jugular veins (IJVs) [2,3,4]. The IJVs, which are the dominant outflow veins from the brain are two collapsible vessels characterized by marked changes in their cross-sectional area (CSA), depending on the transmural pressure on the vessel wall [5]. The overall phenomenon is influenced by the gradient of hydrostatic pressure during the transition from supine to sitting position, and vice versa [6]. Indeed, in supine position the main outflow route is represented by the IJVs [7,8]. Focusing on the brain drainage, it is important to analyze the behavior of the IJVs, which mainly contribute to the blood outflow from the braincase [9,10].

The JVP is one of the main parameters of cardiac status and can be used by cardiologists as a heart-failure parameter [11,12]. It carries information about the physiological status of the heart and possible changes due to loss of functionality, and it is an index of the time variation of the blood volume in the IJV. Blood flow and pressure variations, due to the filling of the right atrium, produce pulsations on the main veins that are transmitted through peripheral veins [6]. These pulsations at the level of IJV produce the so called JVP, which can be detected in a non-invasive way because the IJVs are superficial veins, not protected by bone or cartilage tissues [13]. It has been suggested that the JVP can be used to investigate IJV drainage function and to obtain valuable information about cardiac hemodynamics through the analysis of its waveform [14].

In supine position, the IJV is open, and it represents the main outflow route for the cerebral circulation being able to carry most of blood flow from the brain and from other extracerebral territories [8]. However, the jugular venous system exhibits important flow limitation during upright posture changes. Indeed, the IJVs tends to collapse because of the decrease of transmural pressure due to the gravitational field, causing a significant increase in their resistance [6,15].

**Figure 1:**
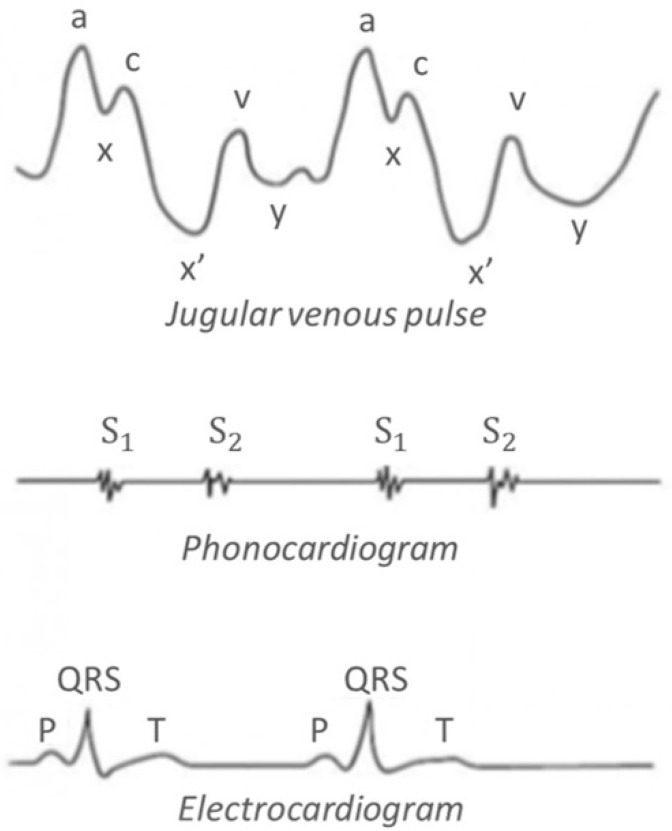
Typical Jugular Venous Pulse wave pattern with the respective sound and electrical synchronization waves.

Figure 1 shows the typical waveforms associated to the vascular pulsations at the level of the neck: it is a normal JVP waveform of an open IJV, associated with a phonocardiogram and an electrocardiogram (ECG) [16].

The JVP pattern consists of 3 ascents or positive waves (*a*, *c* and *v*) and 3 descents or negative waves (*x*, *x*’ and *y*):

1. *a* wave: due to active atrial contraction leading to retrograde blood flow into neck veins.
2. *x* wave: due to continued atrial relaxation, and it coincides with S_1_.
3. *c* wave: due to impact of the carotid artery adjacent to the jugular vein and retrograde transmission of a positive wave in the right atrium produced by the right ventricular systole and the bulging of the tricuspid valve into the right atrium.
4. *x*’ wave: due to descent of floor of right atrium (tricuspid valve) during right ventricular systole and continued atrial relaxation.
5. *v* wave: it coincides with S_2_, and it is less prominent than the “*a*” ascent wave. It corresponds to the maximal atrial filling.
6. *y* wave: it follows S_2_ and corresponds to the emptying of the atrium.

Nowadays, the most used technique to accurately evaluate the JVP waveform concerns central venous line catheterization, but it is an invasive methodology which requires surgery. Other methodologies for evaluating JVP are by means of ultrasounds (US) and in a preliminary experience through near-infrared spectroscopy [2,17]. However, these devices can be bulky and require a specific experimental set-up, which implies the presence of a healthcare operator. Recent non-invasive techniques to evaluate JVP regards the use of plethysmography methodology. Plethysmography is a well-known technique for recording volume changes in a tissue. When the volume change occurs through only a blood volume variation in the tissue, information regarding the tissue blood flow can be obtained by plethysmography.

For the application of this work, such a technique is used to measure variations in electrical signals recorded through a sensor encircling any cylindrical segment of the body. This means that changes in venous volume can be indirectly detected on the skin surface, as displacement [18]. Thus, the aim of this work is to provide a wearable device for plethysmography, based on capacitive strain gauge sensors, which acts locally on the jugular to fully characterize cerebral venous outflow in diagnostics. The wearable device for plethysmography includes three electrodes used to make a synchronized ECG, and a Bluetooth connection to wirelessly transmit the acquired data to a PC for the visualization of the JVP waveform. Section 2 introduces the setup and the experimental activity carried out to validate the proposed device. Then, the collected data will be shown in Section 3 and analyzed in Section 4. Finally, Section 5 summarizes the results of the work.

## 2. Materials and Methods

### 2.1. Capacitive Strain Gauge Sensor

**Figure 2:**
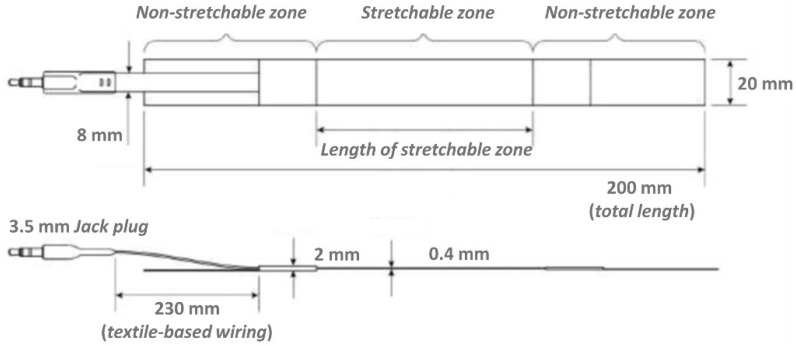
Design and outline dimensions of the capacitive strain gauge sensor.

The capacitive strain gauge sensor chosen for this work is the single layer dielectric electro active polymer (DEAP) PolyPower^®^ film [19], developed by LEAP technology, Aabenraa, Denmark. It is suited to adhere to the skin and record the perimeter variation of the examined anatomic part. It has non stretchable zones at both ends for attachment and a highly stretchable zone as the active part (Figure 2). The output signal represents the elongation of the stretchable zone.

The maximum displacement of a sensor is 80% of the length of its stretchable zone (equivalent to 80% strain). The construction of the sensor is such that in the stretchable zone, a double layer of sensor material is used to make a capacitor with two inner electrodes in the middle and the two outer electrodes on the outside of the sensor. The PolyPower^®^ DEAP material is based on a thin film of elastomer material. The elastomer material is a silicone of the type of polydimethylsiloxane (PDMS), which is a soft material having high dielectric field strength. The basic PolyPower^®^ material has a special micro structured design on the front surface and a flat back (referred to as single layer film material). This single layer film material is used for electrical isolation or as elastomeric film. To get a DEAP material that can be activated, a thin metal electrode layer is deposited on top of the corrugation on the front surface of the single layer film material. Given the micro structured surface, the metal electrode is compliant in one direction across the length of the film web while stiff in the perpendicular direction. The area of the laminated film where the elastomer material is between two electrodes is referred to as the active area. The outer coating of the sensor is made of transparent silicone rubber. For mounting purposes, the sensor is supplied with attachment zones in the two opposite ends. The outer 25 mm at the end of each sensor is a layer of non-woven textile to be used for sewing Velcro stripes useful to close the sensor around a given anatomical section. Regarding the working principle of the strain-gauge sensor (see [20] for details), our plethysmography system uses capacitive sensors for which the capacitance increases when stretched, thus being very responsive to minimal deformations.

### 2.2. Wearable Device for Plethysmography

**Figure 3:**
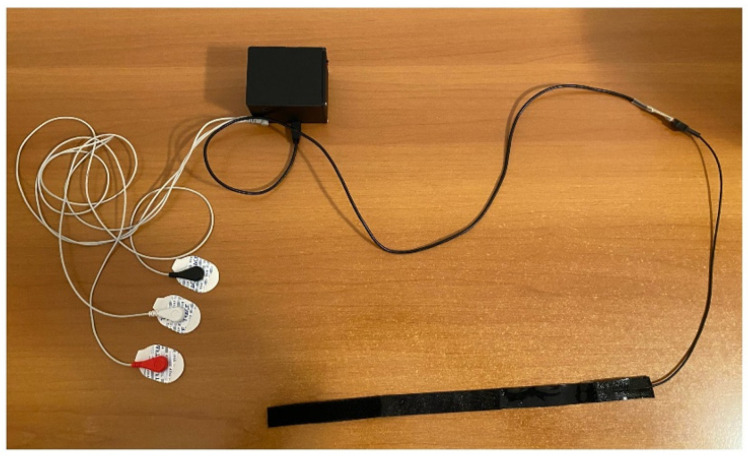
Wearable device for plethysmography.

As shown in Figure 3, the wearable device for plethysmography integrates an electronic unit able to collect data from the capacitive strain gauge sensor, and from the three electrodes used to carry out the ECG measurement. Such a system allows us to perform data transmission for the detailed PC visualization of the JVP waveform together with the ECG trace, synchronously. The electronic unit is compact, i.e., 6.8 cm long, 8.2 cm wide, and 6 cm high, and it is linked to the user interface on a PC through a Bluetooth connection.

### 2.3. US Machine

**Figure 4:**
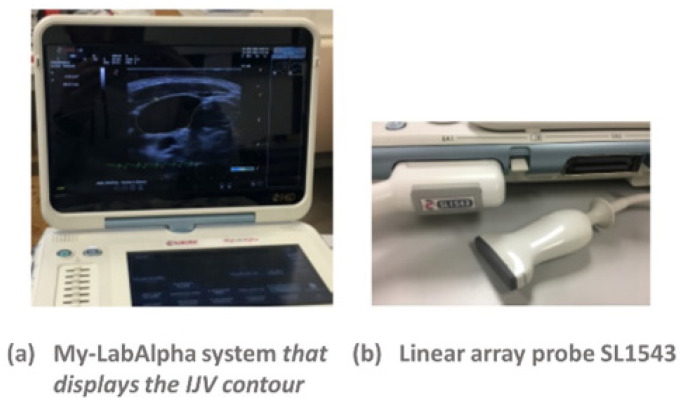
US machine used in this work: My-LabAlpha console (**a**); US probe (**b**).

The US machine used for the scan of the neck is the My-LabAlpha system (ESAOTE), with a linear array probe SL1543 (Figure 4).

Jugular CSA was assessed using a B-mode scan in the transverse plane of the right IJV, in the region that corresponds to the segment close to the junction of the IJV with the subclavian vein [21]. It is better to focus on the right IJV, because it directly communicates with the right atrium via the superior vena cava. Thus, the waveform generated by phasic flow to the right atrium is better reflected on the right side [22].

### 2.4. Experimental Activity

Characterization of both the capacitive strain gauge and ECG sensors was performed before starting the experimental test on subjects.

Regarding the characterization of the strain gauge sensor, two working environments were analyzed:

1. the strain gauge sensor extended and in contact with the air;
2. the strain gauge sensor wrapped around the arm.

The latter configuration is justified by the fact that the sensor is suitable for integral measurements, namely the measurements on the subjects involve the sensor encircled around the neck.

Regarding the characterization of the ECG sensor to verify the frequency response, a pulse generator was used, giving us, as input, a periodic signal. Three fixed values of frequency for the input signal were selected: 0.5, 1, and 2 Hz. The idea is to check if the signal recorded by the ECG sensor changes according to the diverse input signal. For each input signal, the ECG sensor was characterized with the following testing conditions:

1. with the strain-gauge sensor unstretched and in contact with air;
2. with the strain-gauge sensor stretched and in contact with air.

While carrying out the experimental tests on the subjects, the wearable device for plethysmography was applied to the neck to analyze the cerebral outflow in relation to physiological variations, such as breathing and posture. The strain gauge sensor must always be positioned in the same place on the neck at the level of the IJV, adherent to the skin. To avoid as much as possible detection errors, the sensor was placed in the lower part of the neck, closest to the right atrium. This allows us to properly detect the JVP waveform and to avoid as much as possible the artifacts due to the swallowing.

Regarding the ECG measurements, the white electrode was placed on the right side of the chest, just below the collarbone, the black electrode on the left side of the chest, just below the collarbone, and the red electrode was placed on the lower edge of the rib cage, on the left side.

While doing the experiment on subjects, three different postures were analyzed:

1. SUPINE: the subject is in a relaxed position, with arms close to the body, legs extended and with the trunk of the neck parallel to the hospital bed.
2. SITTING: with respect to the supine position, the configuration is the same but with the upper part of the body perpendicular to the legs.
3. UPRIGHT: the subject is perfectly perpendicular to the floor, again with the arms relaxed and parallel to the body.

Again, a simultaneous measurement of the JVP waveform carried out with the US machine and wearable device was performed on a supine subject to preliminarily validate the proposed device.

The study protocol has been approved by the IRBs of the Azienda Ospedaliera Universitaria di Ferrara (Protocol n°. 160499/2016).

## 3. Results

### 3.1. Characterization of the Capacitive Strain Gauge and ECG Sensors

**Figure 5:**
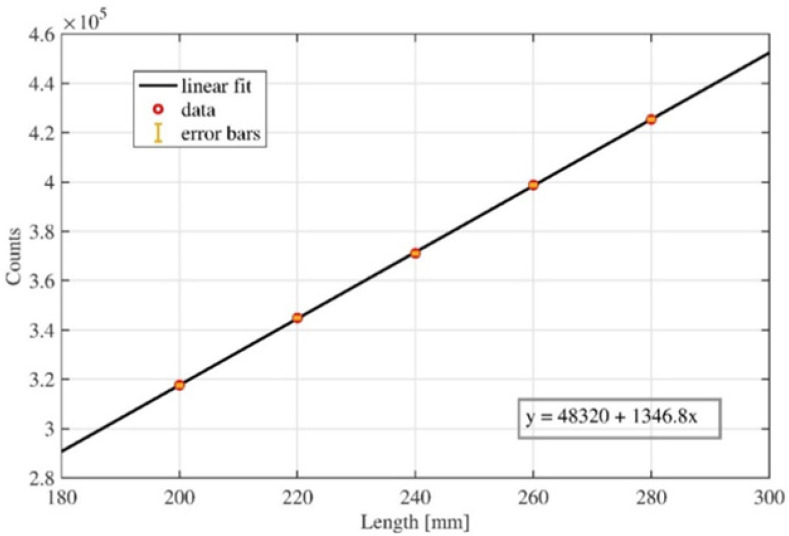
Calibration curve for the capacitive strain gauge sensor. Horizontal axis reports the length of the sensor in mm, vertical axis reports the correlated response of the device in counts, together with its standard deviation.

A calibration curve (Figure 5) was first obtained through the interpolation and approximation of discrete values acquired, making a controlled step by step elongation of the strain gauge sensor. This data acquisition was repeated ten times to evaluate the correlated standard deviation. The obtained values were used to calculate the linear equation together with the *R*^2^ parameter. The value of the *R*^2^ parameter is close to one, so the linear response of the capacitive strain gauge sensor is guaranteed. Measurement accuracy was recorded to be 0.1%.

**Figure 6:**
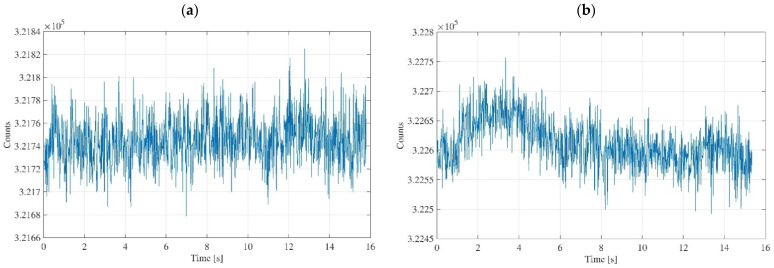
Characterization of strain gauge sensor: for the sensor extended and in contact with air (**a**); for the sensor wrapped around the arm (**b**).

Regarding the characterization of the strain gauge sensor, the data acquisition was initially performed without the subject, and subsequently by wrapping the sensor to the arm, to show the differences in the acquired signals (Figure 6). Concerning the data acquisition without the subject, the sensor was extended and placed on the workbench, putting the active area in contact with air. Figure 6a shows the data acquisition carried out with the sensor extended and in contact with the air, and Figure 6b shows the data acquisition carried out with the sensor wrapped around the arm.

The acquired data were collected in text files, and with MATLAB^®^ software it was possible to develop an algorithm able to calculate the average value of the sensor counts and their trend as a function of time. The data acquisition lasted for about fifteen seconds, and the experimental setup involved disconnecting the PC from the power supply and turning off the Wi-fi to avoid signal distortions due to the environmental noise.

In Figure 6b, it is possible to recognize a slight increase in the average value counts compared to the case in which the sensor is extended and is not applied to any place on the body, Figure 6a. Obviously, this is reasonable because when the sensor is wrapped, elongation of the active area occurs as a result, and this corresponds to an increase in the counts number. This increase is minimal as the elongation is almost imperceptible. Concerning the amplitude of the signal oscillations, it is almost constant, and it is a good information since it means that these oscillations are inherent into the system, and do not depend on the operating conditions. In other words, the typical noise due to the sensor and electronic unit does not change. Rather, it remains constant. Obtaining equal amplitudes for the two signals means that the object is stable from the point of view of the quality of the measurement it produces.

Regarding the ECG sensor, the characterization was carried out using input signals with three different fixed values of frequency: 0.5, 1, and 2 Hz. In this way, we are sure that the input signal covers the electrocardiogram spectrum relative to a bradycardic, physiological, and tachycardic subject, respectively. In the following, Table 1 and Table 2 report the acquired output signal frequency corresponding to the generated input signal frequency for the two different operating conditions: stretched (Table 1) and unstretched (Table 2).

In Table 1 and Table 2, the errors related with the input signals induced by the pulse generator are not reported since the generator is extremely accurate, with a margin of error in the order of 10^−5^–10^−6^. The obtained results are comfortable because they tell us that the ECG sensor operates properly for all the different working settings, with the input signal being perfectly the same as the output one. Therefore, for the experimental tests on subjects, every time we will observe cardiovascular oscillations in the neck, the corresponding ECG will be synchronous.

### 3.2. In-Vivo Plethysmographic Measurements on Healthy Subjects

Measurements were carried out on five healthy subjects, with an average age between twenty-five and thirty-five years. Before starting the experiment, they gave informed consent for carrying out the experiment.

After applying the capacitive strain gauge sensor, and the electrodes in their respective positions to carry out the ECG measurement, we wait about half a minute before starting the data acquisition. This time is needed for a relaxation of the subject. For each subject, three traces of the JVP waveform were acquired corresponding to the three postures: supine, sitting, and upright.

**Figure 7:**
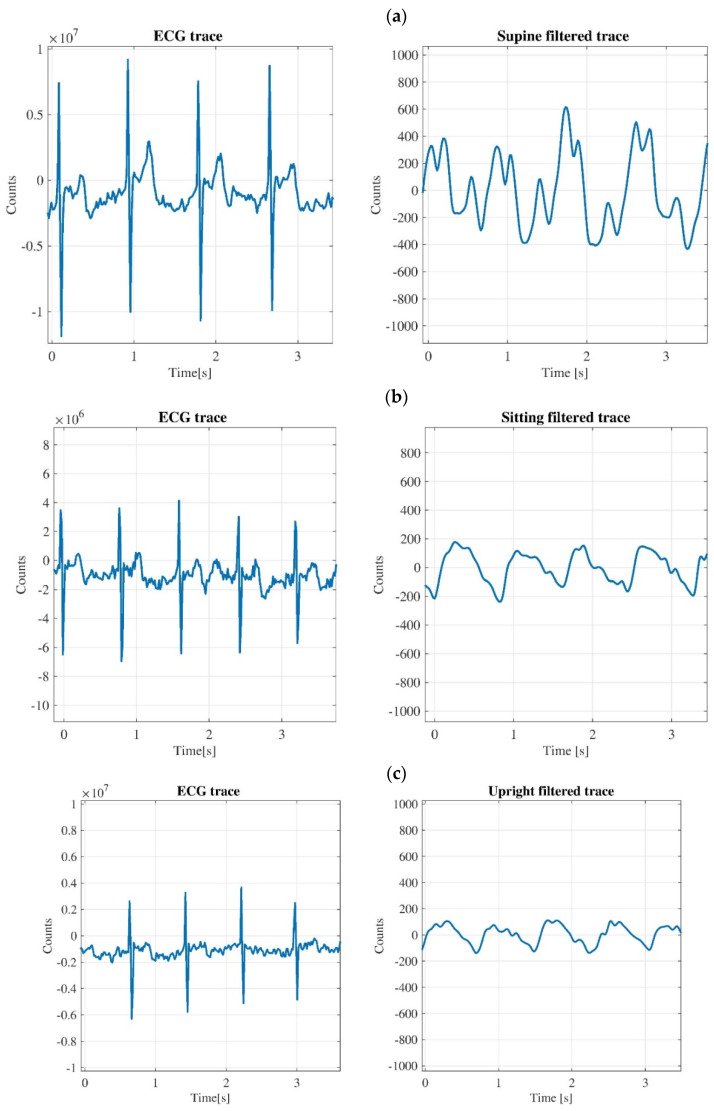
JVP waveform with the corresponding ECG trace for the subject 2 in supine position (**a**); sitting position (**b**); upright position (**c**).

Figure 7 shows the JVP waveforms for the second subjects in supine, sitting and upright position, with the corresponding ECG traces. The pattern of signals in the graphs of Figure 7 are like those obtained for all the subjects. The plethysmography and ECG traces of each subject are shown in Supplementary Figures S1–S4.

Each acquisition lasted for about one minute and there is a time interval of several seconds among measurements for the different postures to allow body fluids and vital parameters of the subjects (heartbeat, breathing) to reach a new equilibrium after the postural change. The reproducibility of the output signal was assessed for each subject before starting the experiment. By visually checking on the monitor the JVP waveform for a period of approximately five minutes, we did not find any variation in the offset and shape of the JVP waveform, apart from the physiological variation due to swallowing.

For each measurement acquired, a text file was created, which contains data related to the sensor counts and data related to the signal acquisition time. The collected data are subsequently analyzed with the MATLAB^®^ software.

For the plethysmography traces the signals are treated with the Wavelet filter, i.e., Wavelet 1-D toolbox, to highlight the cardiac contribution and to remove the breathing one. In the graphs of Figure 7, only a fraction (about three seconds) is shown for both the ECG and plethysmography traces. It is clear to see that the amplitudes of JVP waveform for sitting and upright positions are lower than the amplitude of the trace for the supine case.

As regards Figure 7a, it is possible to affirm that the cardiovascular oscillations taken at the neck level in the supine position are very interesting because the QRS complex of the corresponding ECG is synchronous with the ‘*a*–*c*’ part of the JVP waveform, as it was mentioned in Figure 1. The time interval between peaks ‘*a*’ and ‘*c*’ is around 170 ms, which is a value corresponding to a normal JVP waveform [23].

Concerning the ECG traces for all graphs of Figure 7, it should be noted that these signals are not filtered because in all the acquisitions carried out, the ECG sensor is incredibly performing since the tracks are very clean and regular. Indeed, it is possible to observe the ‘QRS’ complex with high precision and the other two peaks, ‘P’ and ‘T’, are also seen with enough detail.

### 3.3. Ultrasound and Plethysmography Monitoring of JVP

Simultaneous measurements of the JVP waveform with two different instruments, the US machine and the developed wearable device for plethysmography, were carried out to understand if any similarities in the acquired signals are present. Indeed, the US machine is well recognized to be the instrument to monitor the JVP waveform and the goal of this test is to validate our proposed device.

**Figure 8:**
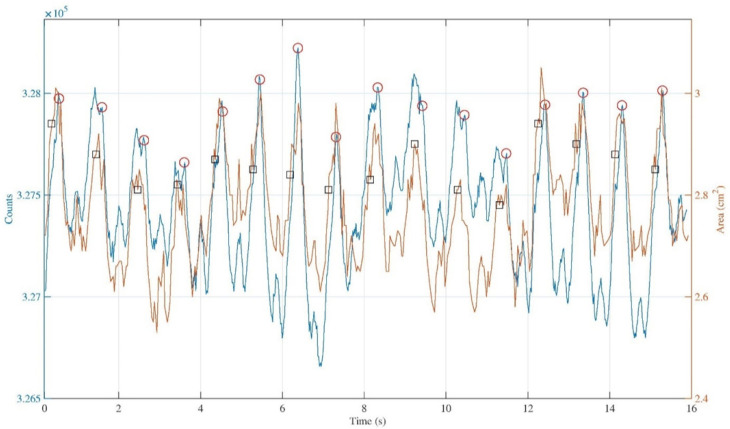
Comparison for the same heartbeats between the JVP waveform (blue) with the corresponding ECG markers (red) and the IJV-CSA signal (orange) with the corresponding ECG markers (black).

The US machine is also equipped with a three-electrode cable for making an ECG measurement. This experiment involved one subject, who gave informed consent before starting the test. The three electrodes of the US machine, and the three electrodes of the wearable device for plethysmography were positioned on the right position of the body to carry out the ECG measurements. The capacitive strain gauge sensor was adapted to the neck, then the US linear probe was used to access at the internal jugular vein. The idea was to synchronize the two ECG traces, in such a way as to have a starting point and an ending point for the synchronization of the two measurements. To obtain this, the subject was asked to perform two Valsalva maneuvers for a few seconds. The Valsalva maneuver was carried out by forcing the patient to exhale against a closed glottis and straining as if he was having a bowel movement [24]. After several seconds, which is the time required for a post maneuver relaxation by the subject, we made a comparison considering about fifteen seconds of the JVP waveform and the corresponding variation of the jugular section for the same heartbeats. The result of the comparison is shown in Figure 8. The IJV contour from the measurement carried out with the US machine, usually called region of interest (ROI), was traced manually for each acquired sonogram.

In Figure 8, the blue and the orange traces are almost comparable, in the sense that qualitatively they show the same periodicity of the signal. The ECG markers in the IJV-CSA signal are not exactly in the same position compared to the ECG markers on the JVP waveform. This could happen since the repeatability of the manual ROI selection operation affects the reliability of the measurement, this operation depends on random errors that can result in under or overestimation of the actual IJV ROI area.

It is possible to make a first quantitative analysis by considering the average delay among ECG markers and ‘*v*’ peaks for all the heartbeats considered in the two different traces. While doing these calculations, a mean value was obtained equal to ∆t_ECG-*v*_ = (0.41 ± 0.02)s for the trace obtained with the wearable device for plethysmography, and equal to ∆t_ECG-*v*_ = (0.61 ± 0.04)s for the trace obtained with the US machine.

**Figure 9:**
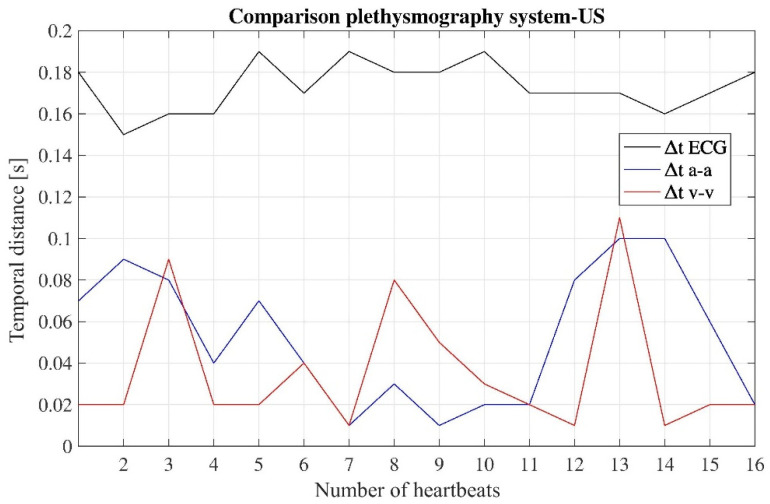
Temporal distances between the peaks ‘*a*-*a*’, ‘*v*-*v*’, and ‘ECG markers’ of the two different signals, for all the considered heartbeats.

Again, a further analysis of the signals shown in Figure 8 was carried out by calculating the temporal distances between the times in which the ‘*a*’, ‘*v*’, and ‘ECG markers’ occur on both the acquired signals. Figure 9 shows the temporal distance of peaks ‘*a*-*a*’, ‘*v*-*v*’, and ‘ECG markers’ between the signal obtained with the developed device and the signal obtained with the US machine, for each heartbeat detected while carrying out the measurement.

From the data in Figure 9, concerning the temporal distances ‘*a-a*’ and ‘*v-v*’, the values do not exceed 0.1 s, while the values of temporal distances ‘ECG markers’ do not exceed 0.2 s. For these data, the resulting mean values are: ∆t*_a_*_-*a*_ = (0.05 ± 0.03)s, ∆t*_v_*_-*v*_ = (0.04 ± 0.03)s, ∆t_ECGmarkers_ = (0.17 ± 0.01)s.

Again, the data summarized in Figure 9 show a linear trend, and the *R*^2^ parameter for the three cases was found to be around 1. Thus, we may assume that the two traces, acquired with the two different instruments, correspond to the measurement of the same physiological phenomenon, namely the JVP waveform over the time.

## 4. Discussion

Nowadays, there is lack of scientific knowledge about many mechanisms involved in human circulation, which are difficult to study because of their high complexity and variability [25,26].

In this research work, it is shown how to analyze cardiovascular oscillations acquired at the neck level using a wireless, non-invasive, wearable device for plethysmography. The goal of the work was to study the feasibility of using the proposed device to easily acquire the JVP signal with the final aim to validate it as a new tool for the clinical investigation of cardiovascular and cerebrovascular diseases. Indeed, the JVP signal carries information about the physiological status of the heart, and it is an index of blood flow variations in the IJV that is the main route for the cerebral venous outflow [27]. Although a 45° thoracic inclination is the gold standard position for the evaluation of the JVP in clinical practice, such a position was not investigated because in this work our aim was to fully characterize the cerebral venous outflow, and the goal of our assessment was the IJV outflow route, which is maximal at 0°. Our group already carried out an investigation of the cerebral Doppler venous haemodynamics of IJV for diverse postural positions (0°, +15°, +30°, +45°) with not significant differences from the 0° position [28]. The measurements for the users in sitting and in upright positions, in which the cerebral outflow is completely re-directed into vertebral veins, were carried out to highlight possible differences in the trace. Thus, it was decided to assess 0° (supine position) as main and 90° (sitting and upright positions) as minor IJV flow routes.

In this early feasibility study, we can affirm that the proposed device is capable to detect physiological variations of blood pulsation considering different postures of the subjects. Particularly, in supine position, the JVP waveform is well recognized, while in the other two positions, the contribution due to the carotid artery is evident because the jugular veins collapse due to the gravitational field when the posture is not supine [7,18]. For the measurements in supine position, the main contribution to the signal is from the IJVs, which are more superficial vessels with respect to the neck arteries. Our future goal is to further refine the data filtering to discern between arterial and venous contributions, e.g., considering the phase in the waveform peaks.

Regarding all the postures assumed by the subjects, the detection of the heartbeat was always correct, and thus the proposed device can be an alternative to the electrocardiography and photoplethysmography systems used for the monitoring of heart rate [29,30,31]. The proposed device can also be used by common end-users as they affirm that heartbeat is the most important bio-parameter that should be transmitted on a body sensor network to check the health status [32].

In order to validate the proposed solution, a comparison was made between the gold standard to measure the JVP, i.e., US machine, and the developed device for plethysmography. As result, similarities in the two traces were found, and therefore the proposed solution is indicated to better investigate the JVP waveform because the acquisition of the JVP signal is faster and requires fewer operator skills. While using the proposed system for plethysmography, the analysis of the JVP waveform is easier, if compared with the extrapolation of the same information through IJV-CSA data, which are obtained by using the US machine. Indeed, the tracking of ROI requires proper skills of the healthcare operator and long operation time.

Regarding the subjects involved in the study, no one suffered from hypertension, and they were all young and healthy as for the investigation of physiological cerebral venous return. As aging, obesity, hypertension, and cardiovascular diseases, among others, influence the JVP waveform, the next step of the research will be the investigation of sub-populations to characterize any JVP waveform, corresponding to diverse classes of population.

The proposed system for monitoring the JVP waveform is a viable alternative to the non-invasive methodologies that are currently under investigation but that still require the presence of a qualified healthcare operator to be carried out, such as using a photoplethysmography imaging system or a microwave sensor, or alternatively via force-coupled single crystal ultrasound [33,34,35,36,37]. Between these methodologies, the photoplethysmography system has seen the greatest advancement in this field by combining multi-site acquisition techniques and machine learning approaches to improve reliability and effectiveness in the assessment of cardiovascular risk [38,39].

There are also epidermal patches sensitive enough to perceive the deformations of the body surface caused by changes in blood flow. Although these patches are now used to mainly monitor the arterial pulse, there are some cases in which researchers investigate the possibility to analyze the JVP waveform through these innovative sensor [40,41]. A possible step of this ongoing research may be the comparison of the proposed device for plethysmography with the epidermal patches, which provide a local measurement directly on the site of the jugular, while the plethysmography returns an integral one since the device encircles the neck.

## 5. Conclusions

This work is based on the development and characterization of a wireless, wearable device for plethysmography. The sensitive part of the device is a capacitive strain gauge sensor that is placed around the neck, like a necklace, to measure the JVP waveform. Moreover, an ECG sensor is also integrated on the wearable device to synchronize the heartbeat with the JVP signal. Measurements on healthy subjects were carried out to study the feasibility of using the proposed device as a tool to monitor cardiovascular parameters, and particularly the JVP waveform. The measurements were carried out by considering different postures for the subjects involved in the experiment. When they were in supine position, the JVP waveform was well recognized, while in sitting and upright positions the contribution due to the carotid artery is dominant and mostly hides the JVP waveform.

The proposed work shows the feasibility of acquiring the JVP signal with a novel device for plethysmography, but the results obtained for its clinical validation are limited because of the low number of subjects and their narrow age range. For the next step of the research activity, there will be a chance to increase the subjects’ database and the possibility to include the analysis of some pathological individuals, in order to study in detail the differences between the JVP waveform of a pathological subject and a healthy one.

## Figures and Tables

**Table 1:** Data acquisition for the ECG sensor when the strain gauge sensor is stretched and in contact with the air.

Input Signal Frequency (Hz)	Output Signal Frequency (Hz)
0.5	0.5 ± 0.003
1	1 ± 0.009
2	2 ± 0.033

**Table 2:** Data acquisition for the ECG sensor when the strain gauge sensor is unstretched and in contact with the air.

Input Signal Frequency (Hz)	Output Signal Frequency (Hz)
0.5	0.5 ± 0.002
1	1 ± 0.010
2	2 ± 0.031

## Supplementary Materials

The following are available online at https://www.mdpi.com/article/10.3390/diagnostics11122390/s1. Figure S1. JVP waveform with the corresponding ECG trace for the subject 1 in supine 18 position (a); sitting position (b); upright position (c); Figure S2. JVP waveform with the corresponding ECG trace for the subject 3 in supine 20 position (a); sitting position (b); upright position (c); Figure S3. JVP waveform with the corresponding ECG trace for the subject 4 in supine 22 position (a); sitting position (b); upright position (c); Figure S4. JVP waveform with the corresponding ECG trace for the subject 5 in supine 24 position (a); sitting position (b); upright position (c).
