# Supplementary material for: Plethysmography System to Monitor the Jugular Venous Pulse: A Feasibility Study
Source: Diagnostics (Basel). 2021 Dec 18;11(12):2390. doi: 10.3390/diagnostics11122390 (PMC8699927; doi:10.3390/diagnostics11122390)
Supplement: Supplementary file 1 [file diagnostics-11-02390-s001.zip › diagnostics-1483905- Supplementary.pdf]

diagnostics

MDPI

Supplementary Materials

Plethysmography System to Monitor the Jugular Venous Pulse: A Feasibility Study

Antonino Proto [1], Daniele Conti [2], Erica Menegatti [3], Angelo Taibi [2,4,*] and Giacomo Gadda [4]

1 Department of Cybernetics and Biomedical Engineering, VSB–Technical University of Ostrava, 17. Listopadu 2172/15, 70800 Ostrava, Czech Republic; antonino.proto@vsb.cz
2 Department of Physics and Earth Sciences, University of Ferrara, Via Saragat 1, 44122 Ferrara, Italy; daniele.conti@edu.unife.it
3 Department of Translational Medicine, Vascular Diseases Center, University of Ferrara, Via Aldo Moro 4, 44124 Ferrara, Italy; mngrce@unife.it
4 Section of Ferrara, National Institute for Nuclear Physics (INFN), Via Saragat 1, 44122 Ferrara, Italy; gadda@fe.infn.it
* Correspondence: taibi@fe.infn.it; Tel.: +39-0532974218

Figures S1–S4 shows the JVP waveforms in supine, sitting and upright position, with the corresponding ECG traces, for the first, third, fourth, and fifth subject, respectively.

Citation: Proto, A.; Conti, D; Menegatti, E; Taibi, A; Gadda, G. Plethysmography System to Monitor the Jugular Venous Pulse: A Feasibility Study. *Diagnostics* **2021**, *11*, x. https://doi.org/10.3390/xxxxx

Academic Editor: Gino Seravalle Seravalle

Received: 12 November 2021
Accepted: 14 December 2021
Published: 18 December 2021

Publisher's Note: MDPI stays neutral with regard to jurisdictional claims in published maps and institutional affiliations.

Copyright: © 2021 by the authors. Licensee MDPI, Basel, Switzerland. This article is an open access article distributed under the terms and conditions of the Creative Commons Attribution (CC BY) license (https://creativecommons.org/licenses/by/4.0/).

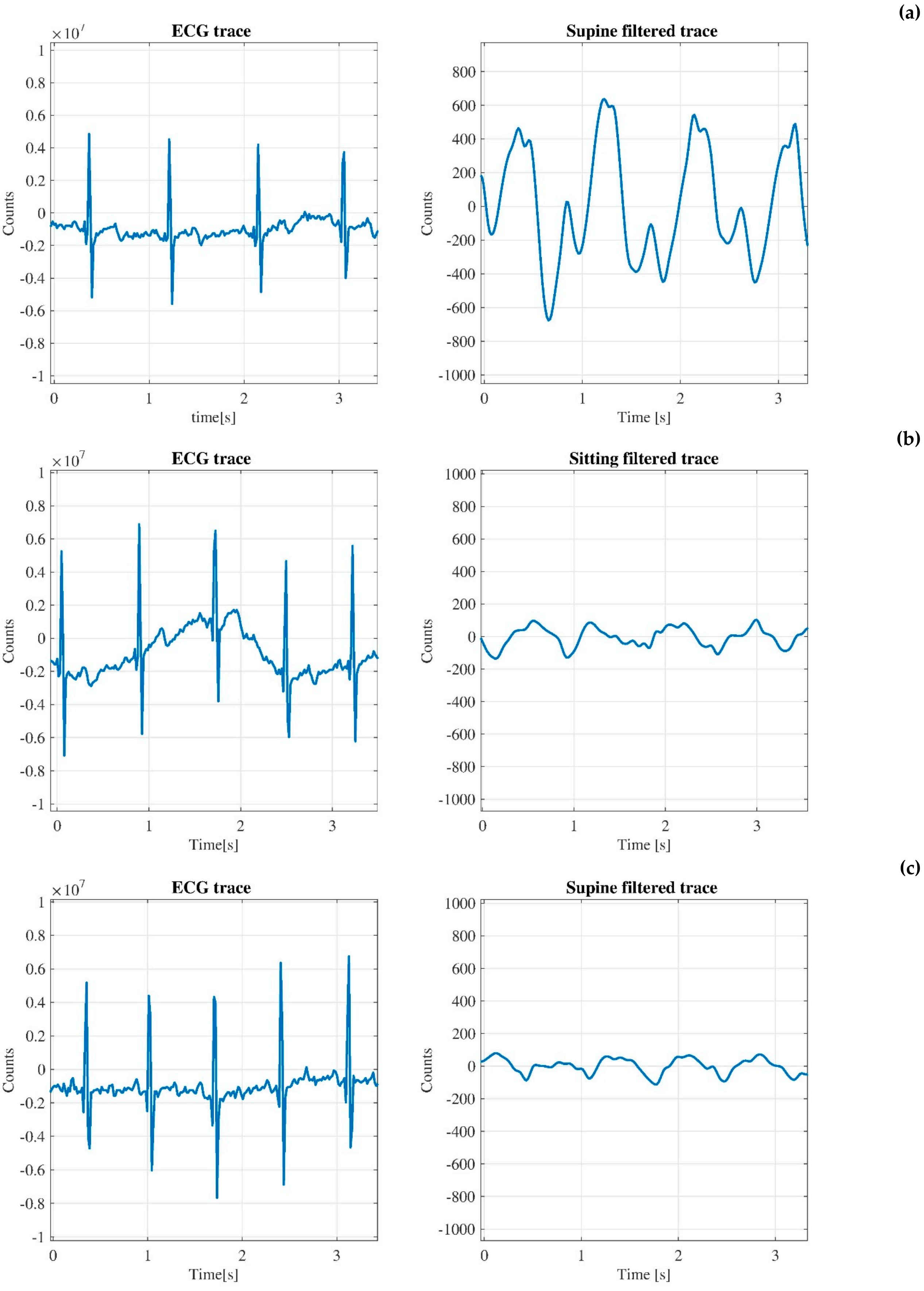

Figure S1. JVP waveform with the corresponding ECG trace for the subject 1 in supine position (a); sitting position (b); upright position (c).

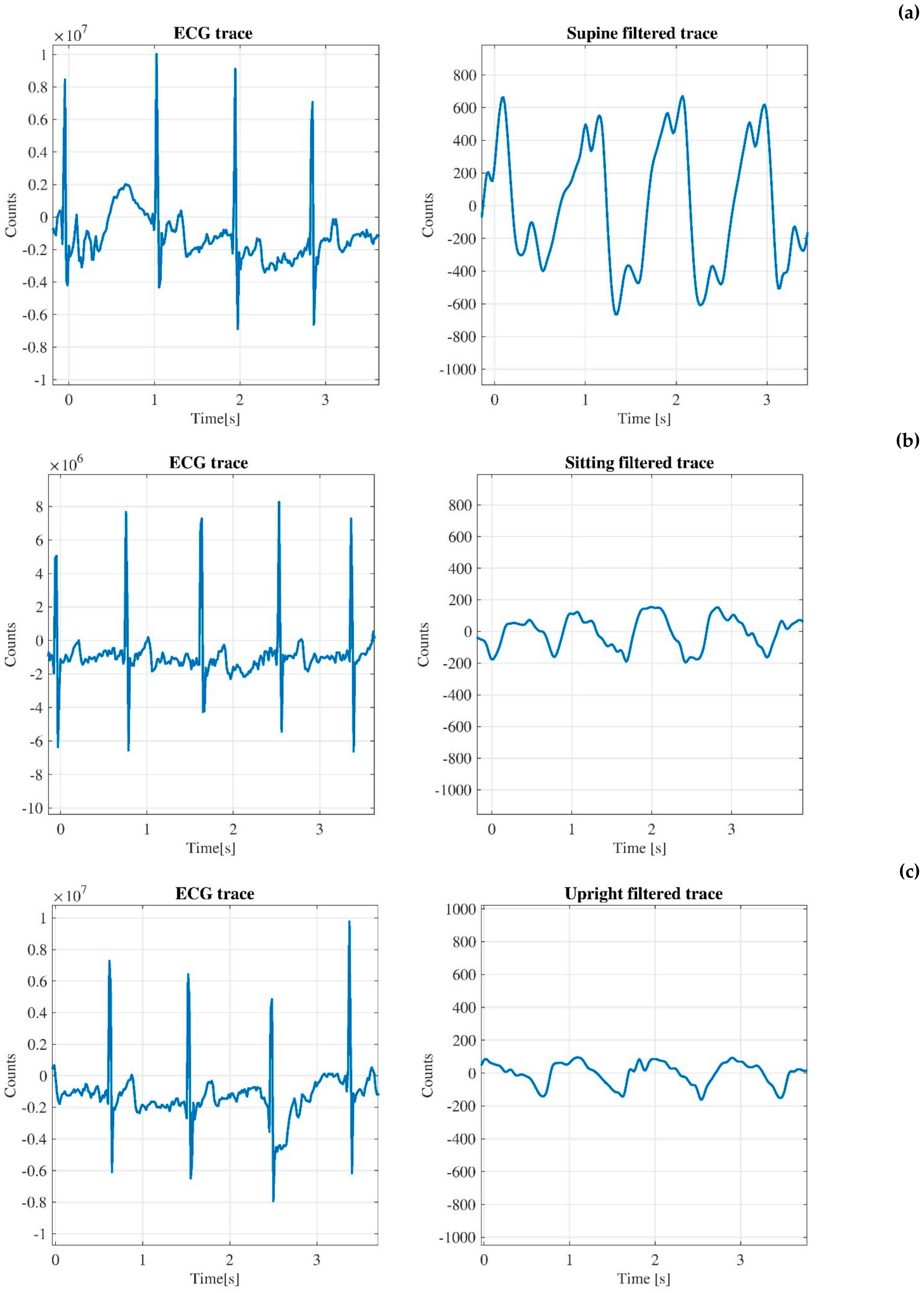
(a)
ECG trace
×10^7
Counts
Time[s]
Supine filtered trace
Counts
Time [s]
(b)
ECG trace
×10^6
Counts
Time[s]
Sitting filtered trace
Counts
Time [s]
(c)
ECG trace
×10^7
Counts
Time[s]
Upright filtered trace
Counts
Time [s]

Figure S2. JVP waveform with the corresponding ECG trace for the subject 3 in supine position (a); sitting position (b); upright position (c).

(a)

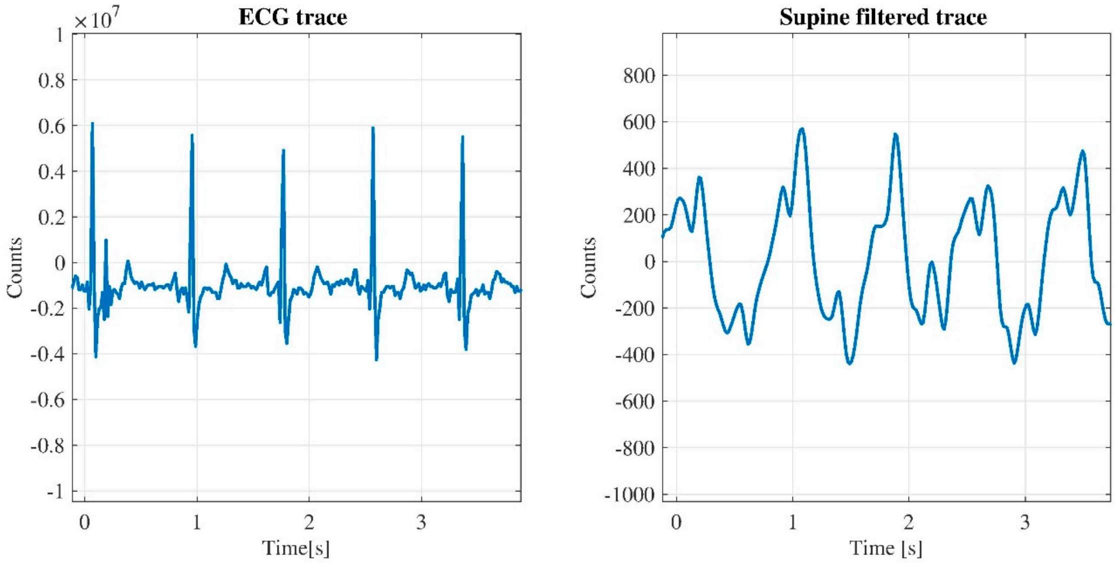

(b)

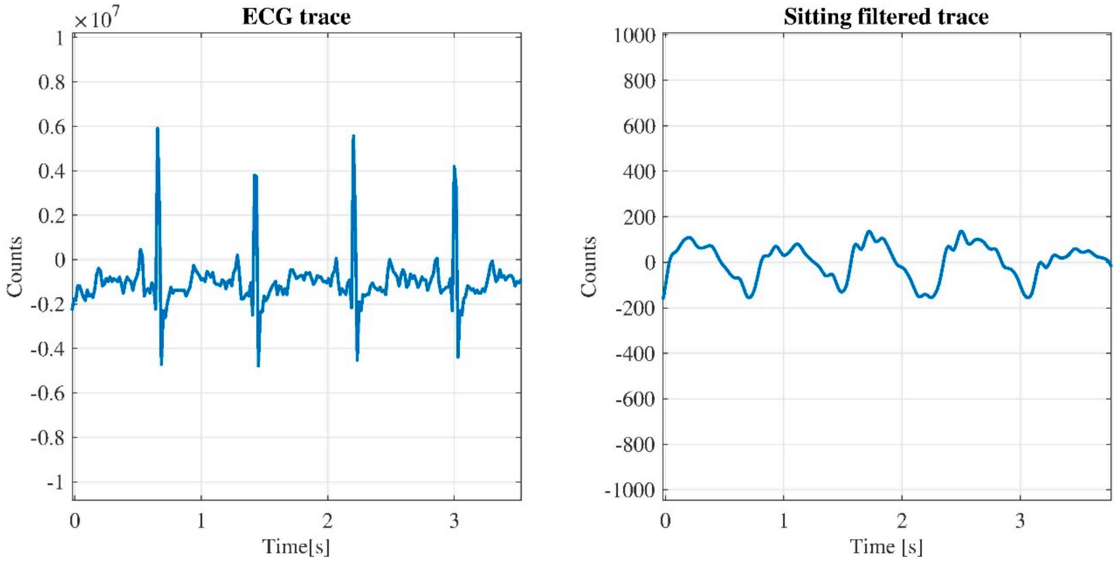

(c)

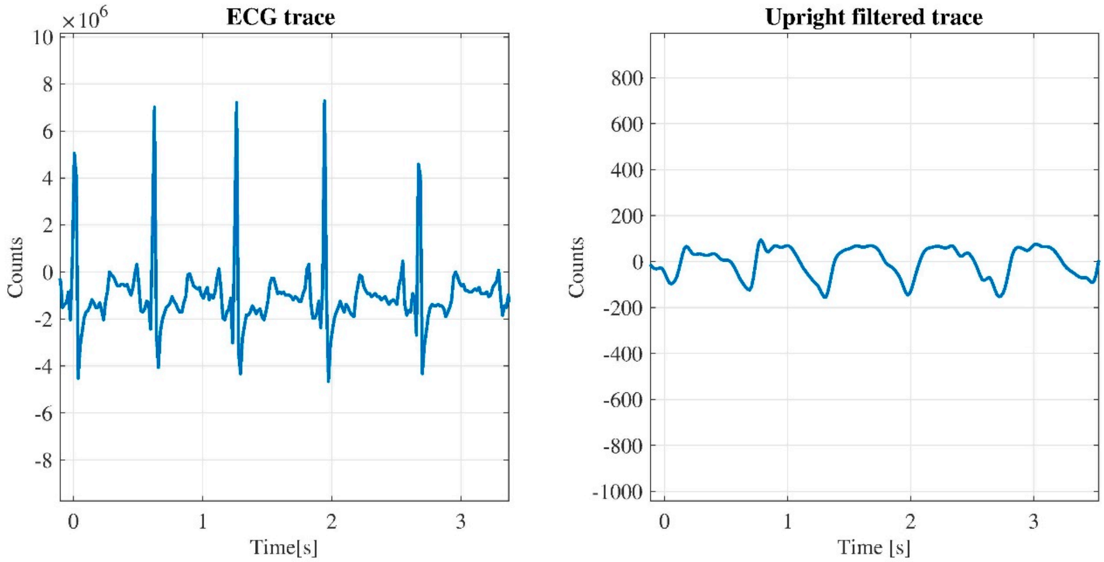

Figure S3. JVP waveform with the corresponding ECG trace for the subject 4 in supine position (a); sitting position (b); upright position (c).

(a)

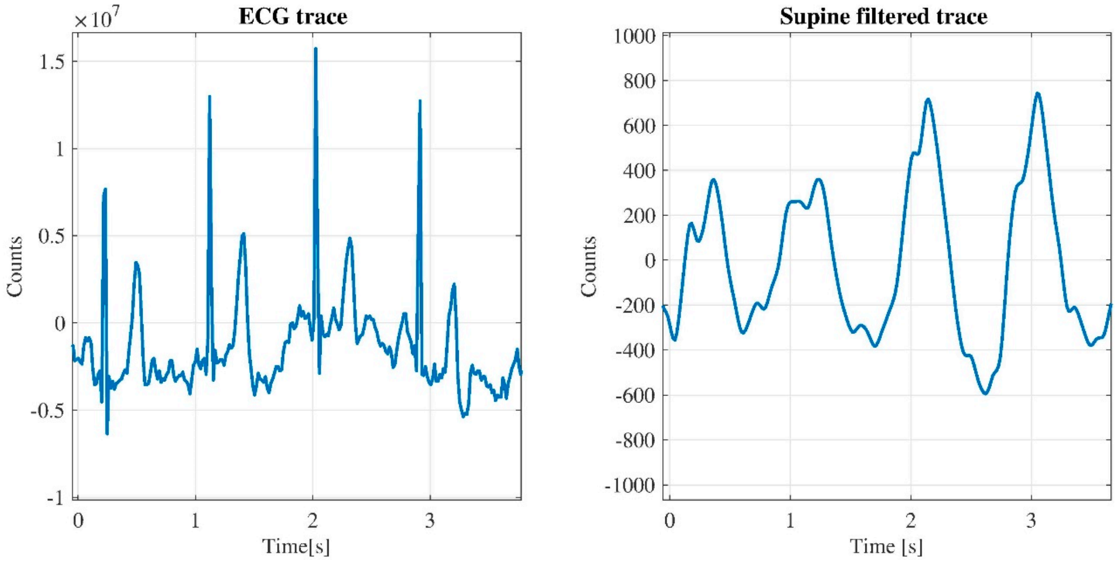

(b)

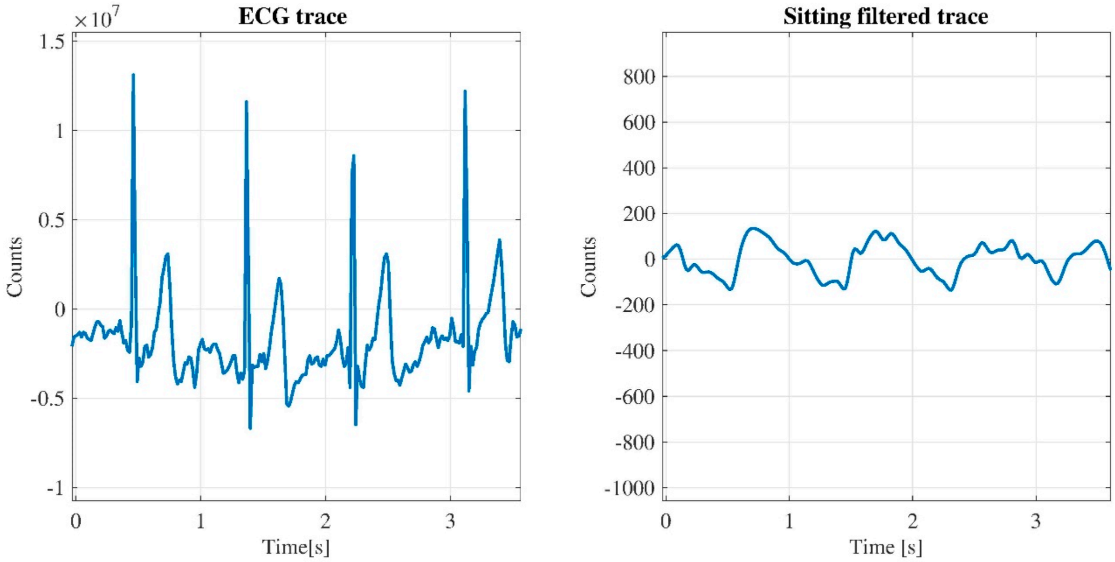

(c)

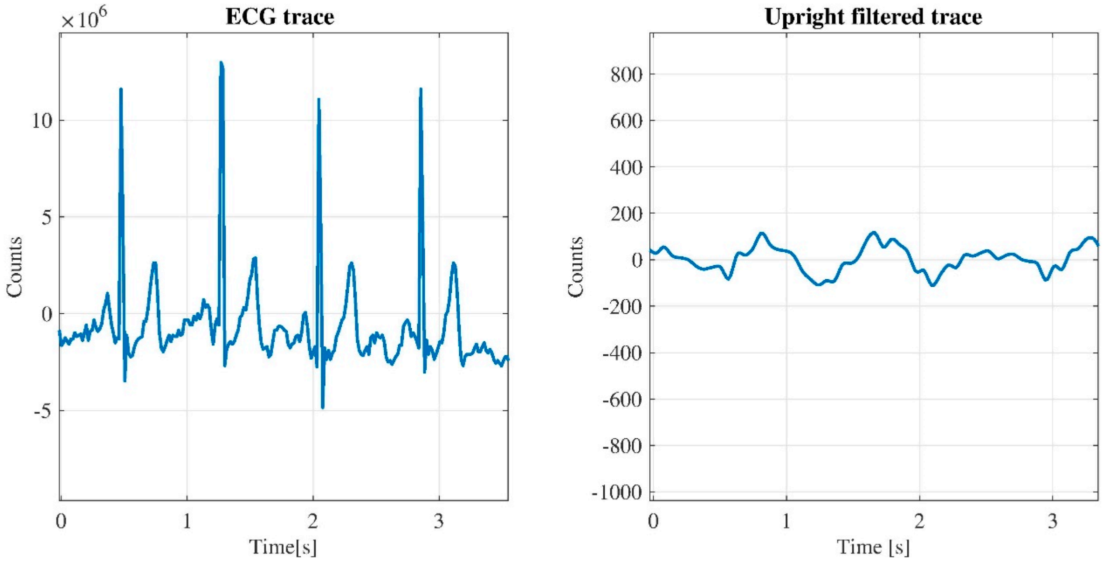

Figure S4. JVP waveform with the corresponding ECG trace for the subject 5 in supine position (a); sitting position (b); upright position (c).
